# Why People Don’t Use Facebook Anymore? An Investigation Into the Relationship Between the Big Five Personality Traits and the Motivation to Leave Facebook

**DOI:** 10.3389/fpsyg.2020.01497

**Published:** 2020-07-10

**Authors:** Seoyeon Hong, Sookwang Klive Oh

**Affiliations:** ^1^Rowan University, Glassboro, NJ, United States; ^2^Pepperdine University, Malibu, CA, United States

**Keywords:** facebook, big five – personality, motivation, social media quitting, leaving facebook

## Abstract

This study linked the big five personality traits with motivational factors to leave Facebook based on a survey of 218 former Facebook users. The big five were related with eight main factors retrieved from existing literature. Results showed that neuroticism was positively related to addiction, banality, peer pressure, and privacy while conscientiousness was negatively related to peer pressure, addiction, annoyance, and emergence of new platforms. Openness was positively related with banality but negatively with addiction and peer pressure. Theoretical and practical interpretations are also discussed.

## Introduction

Facebook has been one of the longest serving, most widely used social media platforms, boasting active users of 2.49 billion and continued growth in advertising revenue (Facebook, 2019). However, practitioners and scholars alike point to the fact that Facebook’s popularity has reached its tipping point and started to “slow down.” For instance, Facebook is losing users in the younger demographic, who are abandoning it for other social media sites, such as Snapchat and Instagram (Lang, 2015; De Veirman et al., 2020). The number of young Facebook users (age between 12 to 34) rapidly declined by almost 20% in 2 years (Edison Research, 2019). This decline may be a significant indicator for the challenging future of Facebook. Simply put, Facebook is no longer the go-to social media for personal and social uses, which can be called a considerable dive given the platform’s popularity over the years. Previous publications have contributed to such decline to the recent Cambridge Analytica scandal (Baer, 2018; Statt, 2018). Despite the incident, users’ main motivation to flee the website appears to be their own (Jagannathan, 2019), which the current paper aims to identify.

It is important to acknowledge that people leave their once favorite social media platform for two reasons. Quitting social media emerges as a popular trend (Rampton, 2019). Studies show that use of social media platforms is strongly associated with symptoms of depression, addiction, and anxiety while it negatively affects mental health and subjective well-being (Merolli et al., 2014; Andreassen et al., 2016; Woods and Scott, 2016; Vannucci et al., 2017). In addition, understanding why this phenomenon occurs would help scholars answer how people will change the way they communicate and behave through online media.

Practically speaking, identifying the motivation to reduce or cease the usage of a major social media platform (e.g., Facebook) holds significant implications. From an individual’s point of view, migrating to new platforms means that the varied interfaces, features, and user base could influence the individual’s attitudes and behaviors both online and off. More broadly, the active public shifts to new platforms (e.g., Snapchat), which becomes crucial for generating and identifying trends or popular ideas. This empirical study specifically focuses on better understanding why Facebook users decide to stop or reduce their use of Facebook associating with psychological traits. In the following sections, we first gathered and organized eight motivational factors (information overload, privacy, banality, addiction, peer pressure, emergence of new platforms, productivity, and annoyance) and incorporated the big five personality characteristics (extraversion, neuroticism, openness, agreeableness, and conscientiousness) to examine if these traits for an individual are correlated with the abovementioned motivational factors and, further, stopping Facebook.

### Motivations for Leaving Facebook

In recent years, Facebook usage has been a dominant research topic in communication as it is the leader in terms of users and revenues. For instance, reasons for using Facebook include self-presentation (Nadkarni and Hofmann, 2012; Tosun, 2012), social capital (Ellison et al., 2007; Valenzuela et al., 2009), usefulness (Lin and Lu, 2011), political participation (Boyd, 2008), and uses and gratification approaches (Joinson, 2008; Urista et al., 2009; Cheung et al., 2011; Smock et al., 2011). However, as “leaving MySpace and joining Facebook was once a transition that young people invest in” (Robards, 2012, p. 394), it is possible that Facebook will meet the same fate in the end. With the fall of this social media pioneer, researchers tried to explain what made people withdraw their attention from certain social media (Ross et al., 2009; Baker and White, 2011), and attempted to identify motivations behind the phenomenon. Such phenomenon, known as “virtual identity suicide” (Stieger et al., 2013, p. 1), calls for empirical evidence to explain why and how it is triggered. Osorio et al. (2017) for the need to identify reasons why people discontinue using a certain social media. To this end, our first research question aims to identify possible factors and motivations that contribute to a user’s decision to leave Facebook **(RQ1).**

Building on the existing literature, we propose eight motives for Facebook withdrawal: *information overload, privacy, banality, addiction, peer pressure, emergence of new platform, productivity, and annoyance* (see Table 1). First, *privacy* is a significant reason to leave Facebook. The concern for privacy is stronger for Facebook quitters than Facebook users (Stieger et al., 2013). Govani and Pashley (2007) also state that personal privacy concerns may strongly impact the decision to participate (or not) on Facebook. For example, in a qualitative study by Dindar and Akbulut (2014), a participant noted that Facebook interferes with privacy too much and another one indicated his/her intention to quit if privacy was violated.

**TABLE 1:** List of motivation factors identified from existing literature.

Motivation factors overlapped	References	Motivation factors finalized
Privacy	Baumer et al., 2013; Rainie et al., 2013; Stieger et al., 2013; Turan et al., 2013; Jordaan and Van Heerden, 2017	Privacy
Cyber safety concerns	Baker and White, 2011	
Self concealing	Dindar and Akbulut, 2014	
Newer platform	Robards, 2012; Salomon, 2013	New platform
Instagram	Brookbank, 2015	
Peer pressure	Robards, 2012; Baumer et al., 2013; Dindar and Akbulut, 2014; Sherman et al., 2018	Peer pressure
Social pressure	Baumer et al., 2013; Stieger et al., 2013	
Banality	Baker and White, 2011; Baumer et al., 2013	Banality
Losing interest	Rainie et al., 2013	
Annoyed/disturbed by others’ posts	Baker and White, 2011	Annoyance
Dislike of other’s self presentation	Rainie et al., 2013	
Too much information	Koroleva et al., 2010	Information overload
Information overload	Maier et al., 2012, 2015	
Addiction	Baumer et al., 2013; Stieger et al., 2013	Addiction
Productivity	Baumer et al., 2013	Wasting time
Wasting time	Borowitz, 2013 Heravi et al., 2017	

Many Facebook users also stopped their activity because of losing interest/banality. Baker and White (2011) interviewed 69 adolescents, and 51% of them said they no longer see interesting content. Participants from Baumer et al. (2013) indicated that Facebook is trivial and uninteresting since they think content on Facebook is no longer entertaining.

Similarly, some research points out that people leave Facebook because they perceive contents to be annoying. They were disturbed by people’s continuous posts and disliked how others presented themselves online (Rainie et al., 2013; Stieger et al., 2013). For instance, a survey from Facebook quitters showed that participants highly agreed that they were annoyed by the unnecessary posts and advertisements (Dindar and Akbulut, 2014).

According to Pew Research Center research (Rainie et al., 2013), 28% of Facebook users said the site has become less important to them. Other scholars showed a similar finding that one of the major risks of using social media is it wastes people’s time (Heravi et al., 2017).

Information overload is a significant contributing factor as well (Koroleva et al., 2010; Maier et al., 2012, 2015). Consuming information is a positive experience until the amount of received information reaches a threshold point. After that, escalating information leads to a decline in processing capacity and results in overload (Miller, 1956). Eppler and Mengis (2008) state that more than enough information available from countless friends on Facebook has caused users to take a break from it. Moreover, a survey study showed that about 6% of Facebook quitters worried about their *addiction* (Stieger et al., 2013). The feeling of getting addicted to Facebook was the main reason for them to leave Facebook (Baumer et al., 2013; Young et al., 2017). *Peer pressure* is also a strong force that influences users’ motivation to leave Facebook (Robards, 2012; Baumer et al., 2013; Dindar and Akbulut, 2014). Ironically enough, users joined Facebook because of their friends but were leaving because of them (Kwon et al., 2013; Turel and Osatuyi, 2017).

Finally, there are many *emerging other social media platforms* available for users (Baker and White, 2011). Appearance of an alternative may be appealing to current users in terms of interests, content, and features among other things. If their friends are starting to use a new social media platform, it is highly likely that people will leave the old platform and join new ones to communicate with them. For instance, Instagram has recorded 900 million active users and is now the fastest growing social media instead of Facebook (Kent, 2017; Sherman et al., 2018).

### Big Five Personality Traits

The big five, otherwise known as the five-factor model, is a framework on the model of personality that contains a universal five factors representing personality traits. The theoretical model is a major predictor of individual behavior, showing that individuals vary in terms of extraversion, neuroticism, openness to experience, agreeableness, and conscientiousness (Costa and McCrae, 1992; Ross et al., 2009; Correa et al., 2010; Stieger et al., 2013). Extraversion is the degree to which a person is sociable and outgoing (Mottram and Fleming, 2009). On the contrary, introverted people find more pleasure in solitary activities; tend to be less open-minded, be less close to others, and have fewer friends; and are more suspicious (Eysenck, 1991; Asendorpf and Wilpers, 1998). Neuroticism refers to a person’s tendency to experience distress and high levels of anxiety that are associated with a sensitivity to threat. It refers to “the extent to which the emotions of an individual vary” (Brown et al., 2002). Conscientiousness refers to the degree of responsibility, orderliness, and precision. It is also considered as being ethical and thorough (Costa and McCrae, 1992). To conscientious individuals, friendships and what others think are less important because they have strict rules for their decision-making process. Openness in people tends to make them creative and curious (Costa and McCrae, 1992). As they are broader-minded and have higher tolerance to different perspectives, they are most likely to use social networking sites to seek out novel experiences. Agreeableness refers to “the general warmth of feelings toward others” (Brown et al., 2002) and reflects friendliness (Costa and McCrae, 1992). Agreeable people are friendly, cooperative, and welcoming (Eysenck, 1991) and have been known to avoid conflict (Wehrli, 2008).

Previous studies show meaningful links between the big five personality traits and several forms of social media usage, including motivation, Facebook posting types, online presentation, and self-representation (Ehrenberg et al., 2008; Ross et al., 2009; Amichai-Hamburger and Vinitzky, 2010; Ryan and Xenos, 2011; Moore and McElroy, 2012; Seidman, 2013; Orchard et al., 2014). Big five personality traits are significantly related with social media activity (Moore and McElroy, 2012). Individuals with high extraversion and openness are more likely to express themselves and post more content on Facebook (Marshall et al., 2015). Kabadayi and Price (2014) argue that extraversion and openness are positively associated with broadcasting intention on Facebook and are negatively associated with communicating purpose. Yet neuroticism negatively predicts broadcasting intentions. Ross et al. (2009) find that higher levels of openness are associated with greater online sociability function use. Marshall et al. (2015) also show that frequency of Facebook posting and self expression are positively related to extravert and openness; communication intention is related with extraversion, openness, consciousness, and agreeableness; and information-seeking behavior is associated with openness, extraversion, agreeableness, and conscientiousness.

Ryan and Xenos (2011) adapted the big five model to see the influence of personality traits to compare Facebook users and non-users. In their study, users were more extraverted although less conscientious than non-users. Ljepava et al. (2013) find that non-users and frequent users are different in terms of social and personality characteristics, which eventually leads to their decisions to utilize Facebook. Such findings confirm that characteristics of non-users can be explained through the big five. While the aforementioned literature mainly focuses on the relationship between the big five and motivation to use social media, the big five model has, in fact, been prominently known for its relationship with various motivation types, including motivation for Internet use (Landers and Lounsbury, 2006), achievement motivation (Bipp et al., 2008), approach–avoidance motivation (Elliot and Thrash, 2002), fundamental motivation (Olson and Weber, 2004), and motivation for academic achievement (Komarraju and Karau, 2005). Therefore, it is plausible to argue that Big Five traits are strongly related to motivation factors regardless of context or direction. Considering that the big five personality traits are related with Facebook use in various contexts—associated with reasons for adoption, usage frequency, indicators for level of activity, communication styles, and tendencies for self-representation—we assume that these personality traits also could be used to adequately predict reasons behind an individual’s decision to discontinue social media use. We aim to synthesize big five personality traits with theoretical frameworks on users’ motives for social media discontinuance and identify any relationships among these factors. To do so, the second research question asks if the five personality traits (extraversion, openness, conscientiousness, agreeableness, and neuroticism) would influence motivations to leave Facebook (RQ2).

## Methods

### Sample

Originally, a total of 503 participants were recruited from the online survey system Amazon Mechanical Turk (mturk). This system is known for its high quality and generalizability of data (Owens and Hawkins, 2018), and researchers confirm that data collected from mturk is demographically diverse (Buhrmester et al., 2011), and provides reliable results that are consistent with decision-making research (Goodman et al., 2013). The IRB approved this study before the data collection, and all participants electronically signed consent forms. Their anonymity was fully protected in the encrypted data file. Since some researchers argue that the quality of mTurk data might be in question (Cheung et al., 2011; Crump et al., 2013), we employed several data-screening and validity-checking steps to ensure the sample is appropriate for the study of social media behavior. First, participants who failed to show having verified Facebook accounts were excluded. Then they were again asked to indicate if they were still actively using Facebook. Second, participants who spent too brief of a time were excluded, accounting for a low 10% percentile. Omitted participants’ average time spent was less than 4 min. Third, data collected from incorrect IP addresses (e.g., outside of the United States) was omitted. Finally, those who were still active Facebook users were excluded. Based on the data-cleaning process, 218 participants were selected for the main analysis to match with the relevant population for the research questions at hand. Participants had 4.7 years of user history (SD = 5.23) before they stopped using Facebook. The sample comprised 68% males (*n* = 148) and 32% females (*n* = 70). The average age of participants was 32 (SD = 9.67), and the biggest ethnicity group was Caucasians, accounting for 54% of the data (*n* = 118).

### Measurements

To identify the motivations for leaving Facebook, one question per each motivation was generated on a 5-point Likert scale [e.g., “I stopped using Facebook because my friends no longer use it (peer pressure),” “I stopped using Facebook because contents are no longer interesting (banality),” “I stopped using Facebook because I switched to a different social media (emergence of new platform)”] (Koroleva et al., 2010; Baker and White, 2011; Maier et al., 2012, 2015; Robards, 2012; Baumer et al., 2013; Turan et al., 2013; Zickuhr, 2013; Stieger et al., 2013; Dindar and Akbulut, 2014). Big five personality traits were measured with 44 items using 5-point Likert scales (John and Srivastava, 1999; Xu et al., 2016): Extraversion (α = 0.92), agreeableness (α = 0.92), openness (α = 0.93), neuroticism (α = 0.91), and conscientiousness (α = 0.91).

## Results

Research question 1 asked what the motivations are for Facebook users to leave. From the results, we compared the means and standard deviations of eight motivation factors identified from the existing literature. Motivation factors with the highest score was the emergence of new platform (*M* = 3.41, SD = 1.30), followed by addiction (*M* = 3.34, SD = 1.28), peer pressure (*M* = 3.25, SD = 1.32), privacy (*M* = 3.19, SD = 1.25), information overload (*M* = 3.06, SD = 1.30), annoyance (*M* = 3.00, SD = 1.25), banality (*M* = 2.61, SD = 1.18), and productivity (*M* = 2.28, SD = 1.19) (Table 2).

**TABLE 2:** Means and standard deviations of motivation factors.

Motivation factors	Item	*Mean*	*SD*
Productivity	I stopped using Facebook because I don’t want to waste time	2.28	1.19
Peer pressure	I stopped using Facebook because my friends no longer use it	3.25	1.32
Addiction	I stopped using Facebook because I am concerned about addiction	3.34	1.28
Banality	I stopped using Facebook because contents are no longer interesting	2.61	1.18
Information overload	I stopped using Facebook because there are too much information	3.06	1.30
New platform	I stopped using Facebook because I switched to different social media	3.41	1.30
Annoyance	I stopped using Facebook because I am disturbed by others’ posts	3.00	1.25
Privacy	I stopped using Facebook because I don’t want others to see my information	3.19	1.25

To answer research question 2, correlation analyses were used to test relationships between the aforementioned motivation factors to leave Facebook and the big five personality traits (Table 3).

**TABLE 3:** Means, standard deviations, and Pearson’s correlations among variables.

	1	2	3	4	5	6	7	8	9	10	11	12	13
1. Extraversion													
2. Agreeableness	0.24**												
3. Conscientiousness	0.32**	0.62**											
4. Neuroticism	0.42**	−0.53**	−0.56**										
5. Openness	0.20**	0.37**	0.42**	−0.14*									
6. Wasting time	−0.17*	–0.07	–0.01	0.13	0.12								
7. Peer pressure	0.08	0.17**	−0.18**	0.08	−0.18**	0.05							
8. Addiction	–0.04	−0.21**	−0.25**	0.17*	0.19**	0.06	0.23**						
9. Banality	–0.08	–0.01	–0.05	0.14*	0.14*	0.43**	0.25**	0.11					
10. Information overload	–0.01	–0.10	−0.21**	0.15*	–0.04	0.20**	0.19**	0.43**	0.32**				
11. New platform	0.14*	–0.11	−0.17*	–0.00	–0.13	–0.05	0.44**	0.36**	0.00	0.16*			
12. Annoyance	–0.11	–0.04	−0.18**	0.22**	–0.06	0.18**	0.06	0.32**	0.15*	0.29**	0.15*		
13. Privacy	0.29**	−0.23**	−0.27**	0.26**	–0.05	0.24**	0.14*	0.14*	0.22**	0.25**	–0.02	0.18**	
Mean	3.06	3.56	3.66	2.72	3.52	3.72	2.75	2.66	3.39	2.94	2.59	3.00	2.81
SD	0.83	0.74	0.75	0.90	0.68	1.19	1.32	1.38	1.18	1.30	1.30	1.25	1.25

Significant level at p < 0.01** and < 0. 05*.

Specifically, extraversion was positively associated with the motivation factor of emergence of new platform (*r* = 0.144, *p* < 0.05), negatively associated with privacy (*r* = −0.288, *p* < 0.01), and waste of time (*r* = −0.172, *p* < 0.05). This implies that extraverted people could be more motivated to leave Facebook due to a new platform. Neuroticism was positively related to five motivation factors for leaving Facebook, including addiction (*r* = 0.168, *p* < 0.05), banality (*r* = 0.143, *p* < 0.05), annoyance (*r* = 0.218, *p* < 0.01), privacy (*r* = 0.260, *p* < 0.01), and information overload (*r* = 0.145, *p* < 0.05). This shows that neurotic individuals are motivated to quit Facebook when they are annoyed by content, when there is too much information, when they no longer see interesting content, and when they are concerned with addiction and privacy. Openness was positively related with banality (*r* = 0.135, *p* < 0.05) while negatively associated with addiction (*r* = −0.191, *p* < 0.01) and peer pressure (*r* = −0.175, *p* < 0.01). In this regard, individuals who scored higher on the trait of openness are more susceptible to boring content, yet they are less likely to be motivated by their friends and addiction concerns when they are set to leave.

Conscientiousness was negatively related to six motivation factors, including peer pressure (*r* = −0.180, *p* < 0.01), addiction (*r* = −0.249, *p* < 0.01), annoyance (*r* = −0.183, *p* < 0.01), emergence of new platform (*r* = −0.172, *p* < 0.01), information overload (*r* = −0.211, *p* < 0.01), and privacy (*r* = −0.265, *p* < 0.01). Finally, agreeableness was negatively associated with peer pressure (*r* = −0.173, *p* < 0.05), addiction (*r* = −0.206, *p* < 0.01), and privacy (*r* = −0.230, *p* < 0.01). This indicates that agreeable individuals are less motivated by privacy concerns and addictions issues. Also, they are less influenced by the lack of their friends being on Facebook.

## Discussion

The present research explores factors that drive users to leave Facebook and how they are related to big five personality characteristics. It is important for scholars to understand the phenomenon underlying people’s departure from the biggest social media platform. Facebook has been extremely influential in society due to its sheer reach and high levels of user interaction. If such engagement decreases due to users leaving the platform and/or switching to different platforms, the social media communication landscape will be reshaped a great deal. This study contributes to a better understanding about how users with different personality traits might play a role in this transition. Moreover, findings from this study could shed light on how social media platforms, as a communications service, could cater to different users better.

Our research question 1 identifies eight motivation factors for Facebook withdrawal: *information overload, privacy, banality, addiction, peer pressure, emergence of new platforms, productivity, and annoyance*. A major contribution is that we supplement the needs from research attempting to identify primary reasons to quit social media. To fulfill this gap, we adapt the big five personality traits to predict those motivation factors. Each individual possesses his or her own level of traits in the big five as the traits are known to be universal. However, given that the variation of traits greatly differ, we aim to utilize this association in explaining who has the tendency to become a social media vagabond.

Findings from research question 2 show that neuroticism predicts motivational factors to leave Facebook. Neurotic individuals stop using Facebook because of privacy concerns, addiction concerns, banality of content, annoying content, and information overload. This finding is consistent with previous literature indicating that neurotic people prefer to control what information is shared (Ross et al., 2009) and are anxious about self-presentation, which leads to social anxiety (Trapnell and Campbell, 1999). They only use Facebook when they are convinced that it is a safe place for safe expression (Forest and Wood, 2012). Not only do they not want to receive too much information, but they also do not want to express too much on Facebook. Therefore, privacy could be a big issue for Facebook quitters who have high neuroticism. On the other hand, individuals with low neuroticism broadcast more content on Facebook (Kabadayi and Price, 2014).

Motivation about content banality can be mainly explained with openness. It is not surprising that more open individuals do not wish to spend their time watching boring content. Individuals with high openness scores may be inclined to trying new methods of communication or using a new platform to gain novel experiences (Butt and Phillips, 2008). Openness is positively correlated with greater social media use in early stages (Correa et al., 2010) and posting more on others’ walls (Ross et al., 2009). Moreover, they share more personal information on Facebook profiles and are more self-disclosing (Guadagno et al., 2008; Amichai-Hamburger and Vinitzky, 2010). This indicates that people who are open-minded want to experience more and are more flexible (McCrae and Costa, 1991; Madjar, 2008).

Extraverted people leave Facebook because of new platforms while introverted people are influenced by concerns about privacy and productivity. This is the only personality trait that is positively associated with the introduction of a new platform. This is consistent with previous findings showing that a high level of extraversion is related to active self-presentation and self-consciousness (Trapnell and Campbell, 1999; Seidman, 2013). Practically speaking, this trait could have the strongest influence when users leave Facebook for newer platforms, such as Instagram or Snapchat. With the increasing popularity of those two platforms and the possibility of more to come in the future, it is important to acknowledge what extraversion could predict in terms of switching to other social media platforms.

In addition, our findings show that less agreeable individuals stopped using Facebook because of privacy concerns and addictions issues. As individuals who have high scores on agreeableness are good minded and trustful, it is reasonable to assume that less agreeable individuals are more suspicious, which could explain their concerns about addiction and privacy. It is also known that less agreeable people are usually uneasy and censorious (Landers and Lounsbury, 2006; Butt and Phillips, 2008). Conscientiousness is positively related to the quality and quantity of interpersonal relationships (Asendorpf and Wilpers, 1998). This explains why our data shows that less conscientious individuals were likely to be influenced by their peers and they want to follow them to new social media platforms in their decision to leave Facebook. Conscientious people are dutiful and responsible; they do not want to simply quit while there are still relationships remaining through Facebook. Additionally, conscientiousness was negatively associated with social media use (Wilson et al., 2010; Ryan and Xenos, 2011), and the most salient predictor of self-representation: Conscientious individuals are more cautious.

Interestingly, privacy concerns appear to be related to four personality traits out of five: extraversion, agreeableness, consciousness, and neuroticism. Interestingly, Ljepava et al. (2013) state that openness is related to privacy. However, privacy concerns were not related to openness in our data. This may indicate that the issue of privacy has become a universal concern for social media users in general. Govani and Pashley (2007) argues that personal privacy significantly impacts their decision on Facebook usage. This relates to a meaningful interpretation in regard to the Cambridge data breach scandal. According to our results illustrating a strong association between privacy concerns and personality traits, a strong privacy protection policy seems an inevitable choice for Facebook. In this regard, our findings also provide some invaluable implications for practitioners and social media users.

First, practitioners who engage in social media planning and strategies can benefit from carefully considering factors that may lead to users’ willingness to leave the platform. From platforms’ perspectives, practitioners ought to assess the status and characteristics of the communication channel, forming strategies based on how users may react because they are ready to switch to the next platform, and our findings can be further applied to other social media platforms. As people are not leaving social media but leaving Facebook, it is safe to say they are just shifting between platforms. As motivation factors integrated in our study were relevant to social media usage in general, our findings can be replicated with other social media platforms once they reach a tipping point in terms of popularity and usage.

Second, from social media users’ perspectives, understanding the relationship between motivation and personality is particularly important because personality traits are intrinsic (Ljepava et al., 2013) and cannot be easily changed. People’s personality characteristics are significantly associated with social media use (Correa et al., 2010; Błachnio et al., 2016). For instance, concerns (e.g., annoyance) deriving from personality traits cannot be controlled; rather, practitioners should focus on minimizing other factors (i.e., in and of the platform) that may lead to the willingness to stop using social media altogether. It is noteworthy that searches for “delete Facebook” are strongly correlated with terms like “being happy”. This indicates individuals are negatively affected given that the more time they spend on Facebook, the worse (Kross et al., 2013), and more lonely (Pittman and Reich, 2016) they feel. Instead of feeling “bad” or “lonely,” individuals could control and manage their feelings if they know how the motivation factors are related with their personality.

We acknowledge that the current study has some limitations that can be addressed in subsequent studies on this topic of research. First, each motivation factor was measured with a single item. Even though single-item usage is common in measuring relatively new factors (Correa et al., 2010; Maier et al., 2012, 2015), the future study should develop the new measurement tested by factor analysis to build more reliable items for motivations. Second, we did not distinguish between temporary quitters and eternal quitters. According to Schoenebeck (2014), eternal quitters and temporal break-takers differ in their motivations. Quitters seek to make long-term changes while break takers seek short-term changes. A recent study (Rainie et al., 2013) showed that 61% of Facebook users have taken a voluntary break from the site, but it did not explain whether they became eternal quitters. End users should be considered differently from social media users who change platforms periodically in the future study. Our study is rather limited in scope to Facebook. The motivation factors may not be applicable for other and relatively new social media platforms, such as Instagram or Snapchat. There are more starters, and not many quitters yet. The motivation factors we’ve identified for this study were initially developed and tested when users started to deviate from Facebook. Facebook was already in the maturation stage during its life cycle. There is, thus, a need for future studies focusing on user motivations for quitting in the context of other social media platforms.

## Data Availability Statement

The datasets generated for this study are available on request to the corresponding author.

## Ethics Statement

The current study involving human participants was reviewed and approved by IRB. Authors received electronic consents from participants.

## Author Contributions

SH – bibliography, study design, data collection, and analysis. SO – bibliography, data collection, and manuscript writing. Both authors contributed to the article and approved the submitted version.

## Conflict of Interest

The authors declare that the research was conducted in the absence of any commercial or financial relationships that could be construed as a potential conflict of interest.
